# Cytokine profiles and diagnoses in elderly, hospitalized psychiatric patients

**DOI:** 10.1186/s12888-018-1900-y

**Published:** 2018-09-27

**Authors:** Erlend Bugge, Rolf Wynn, Tom Eirik Mollnes, Solveig Klæbo Reitan, Ole Kristian Grønli

**Affiliations:** 1 Division of Mental Health and Addictions, University Hospital of North Norway, N-9037 Tromsø, Norway; 2 Department of Clinical Medicine, UiT The Arctic University of Norway, N-9038 Tromsø, Norway; 3 UiT The Arctic University of Norway, K.G. Jebsen TREC, N-9038 Tromsø, Norway; 4 Research Laboratory, Nordland Hospital, Bodø, Norway; 5 Faculty of Health Sciences, K.G. Jebsen TREC, University of Tromsø, Tromsø, Norway; 6 Department of Immunology, Oslo University Hospital, and University of Oslo, Oslo, Norway; 7 Centre of Molecular Inflammation Research, Norwegian University of Science and Technology, Trondheim, Norway; 8 Norwegian University of Science and Technology, Faculty of Medicine and Health Sciences, N-7491 Trondheim, Norway

**Keywords:** Psychogeriatric, Gerontopsychiatric, Cytokine, Depression, Neuroimmunology

## Abstract

**Background:**

There is a paucity of studies on inflammatory markers in elderly psychiatric patients. Hence, our study was undertaken to investigate cytokines as biomarkers in diagnostically unselected elderly patients admitted to a psychiatric hospital.

**Methods:**

Demographic data, clinical data and blood samples, including 27 cytokines, were collected from 98 patients above 60 years, consecutively admitted to a psychiatric hospital in Tromsø, Norway (69°N).

**Results:**

The most common diagnosis was Recurrent depressive disorder (26.5%), the second most common was dementia in Alzheimer’s disease (20.4%). The most frequent somatic disease was cardiovascular disease (28%).

No statistical association (*p* < 0.01) was found between cytokines and gender, age, BMI, anti-inflammatory drugs, psychotropic drugs, reason for admittance, smoking, vitamin supplements, alcohol consumption, length of stay, somatic disease (present/not-present) or psychiatric diagnoses. However, when allocating patients to two groups, *depression* and *no depression*, we found higher levels of 10 cytokines in the *no depression* group (FDR-*p* < 0.0044). Possibly, this could in part be explained by the higher prevalence of cardiovascular disease (CVD) and dementia in the no depression group, as these factors were significant predictors of patients being categorized as not depressed in a logistic regression. In addition, other unknown factors might have contributed to the association between no depression and elevated cytokines. On the other hand, the high level of psychiatric and somatic comorbidity in the study population may have led to increased levels of cytokines in general, possibly diluting the potential effect of other factors, depression included, on the cytokine levels.

The size of the study, and particularly the size of the subgroups, represents a limitation of the study, as do the general heterogeneity and the lack of a control group.

**Conclusions:**

There was no significant difference in cytokine levels between various psychiatric diagnoses in hospitalized elderly psychiatric patients. This indicates that previous findings of correlations between cytokines and various psychiatric disorders in highly selected adult cases might not be applicable to elderly psychiatric inpatients.

Further immunological studies are needed on gerontopsychiatric patients in general and gerontopsychiatric patients with specific disorders, preferably with patients that are physically healthy.

**Trial registration:**

Retrospectively registered in the ISRCTN registry study, with study ID ISRCTN71047363.

## Background

Several studies have demonstrated an association between psychiatric disorders and biomarkers of inflammation, particularly cytokines. Primarily, these studies have focused on specific disorders and selected sets of cytokines. Depression and schizophrenia seem to dominate this research, both disorders repeatedly demonstrating elevated levels of pro-inflammatory cytokines such as IL-1β, IL-2, IL-6, and TNF [1–3]. Though some of these studies include elderly patients [4–6], most studies have been conducted on younger adults. Thus, there is a paucity of studies on elderly psychiatric patients and particularly elderly psychiatric in-patients. Besides the fact that populations are ageing in most countries [7], the elderly are of particular interest because they are more likely to represent biological diversity due to age-related neuroimmunological changes [8] and higher frequencies of comorbid conditions. Consequently, findings of cytokine changes in younger adults do not readily translate to elderly psychiatric patients. Hence, our study was undertaken to investigate cytokines as biomarkers in diagnostically unselected elderly patients admitted to a psychiatric hospital.

## Methods

### Population

The population has been described in a previous publication [9]. Demographic data, clinical data and blood samples were collected from 98 patients, 60 years and older, consecutively admitted to a psychiatric hospital in Tromsø, Norway (69°N). The catchment area of the hospital was approximately 250,000 citizens. Exclusion criteria comprised inability to communicate and cooperate, e.g. due to a severe psychiatric condition like severe dementia or confusion/delirium, or a medical condition likely to significantly affect the blood/plasma analysis like severe dehydration or ongoing infection. The reasons for referral included a variety of psychiatric conditions, spanning from anxiety to psychosis, with depression (42%) and dementia (26%) being the most common. In terms of gender, age and diagnostic distribution, the study population was quite similar to the general population of patients admitted to gerontopsychiatric units in Norway [10], the possible exception being a lower proportion of dementia. However, the Norwegian national data included patients with severe dementia, whereas these patients were excluded from our study.

### Clinical assessment

The following instruments were applied to assess the psychiatric and cognitive status of the participants (N = number of patients): the MINI International Neuropsychiatric Interview, *N* = 43 [11], the Montgomery and Aasberg Depression Rating Scale, *N* = 76 [12], the Cornell Scale for Depression in Dementia, *N* = 22 [13], the Mini-Mental State Examination, *N* = 92 [14] and the Clockdrawing Test, *N* = 90 [15]. In addition, clinical interviews and reviews of medical records were undertaken by experienced clinicians in assessment and diagnostics, according to ICD-10 research criteria. Interview of next of kin was also undertaken when appropriate.

### Blood samples

During the first 3 days of admittance, morning blood samples (before 10 AM) were obtained for a range of analyses, e.g. electrolytes, liver enzymes, blood cells and thyroid hormones. In addition, plasma samples from EDTA-tubes were successively and rapidly frozen to − 70 °C, until analysed for cytokines in one batch. The analyses were performed by multiplex technology on a Multiplex Analyser with a predefined kit, according to the instructions of the manufacturer (Bio-Plex Human Cytokine 27-Plex Panel; Bio-Rad Laboratories Inc., Hercules, CA, USA). The assay was set up to detect the following interleukins, chemokines and growth factors: IL-1β, IL-1 receptor antagonist (IL1-ra), IL-2, IL-4, IL-5, IL-6, IL-7, IL-8, IL-9, IL-10, IL-12 (p70), IL-13, IL-15, IL-17, eotaxin, basic fibroblast growth factor (bFGF), granulocyte-colony stimulating factor (G-CSF), granulocyte macrophage colony stimulating factor (GM-CSF), interferon (IFN)-γ, interferon-inducible protein (IP-10), monocyte chemotactic protein (MCP-1), macrophage inflammatory protein (MIP-1α, MIP-1β), platelet derived growth factor-BB (PDGF-BB), regulated upon activation T cell expressed and secreted (RANTES), tumor necrosis factor (TNF), and vascular endothelial growth factor (VEGF).

GM-CSF and IL-15 had a high frequency of non-detectable levels, i.e. below the lower detection limit, and were therefore excluded in the statistical analyses. Another eight cytokines had a small number of patients with cytokine levels below the lower detection limit (number of patients with non-detectable levels): IL-2 (4), IL-10 (11), IL-13 (1), IL-17 (4), bFGF (2), G-CSF (2), PDGF-BB (2), and VEGF (4). Data for these patients were imputed using SPSS, see Statistical analyses section.

### Statistical analyses

Most of the data were not strictly normally distributed, as demonstrated by the Kolmogorov-Smirnov test, and several groups had unequal variances. Thus, nonparametric tests were applied. The Spearman rank correlation coefficient and Kendall Tau coefficient were used to analyse differences between the rankings of two variables. The Mann-Whitney U or the Kruskal-Wallis tests were applied when comparing ranks of two or several subgroups, subsequently. Goodness of fit was assessed by binary logistic regression. To examine whether the raised cytokine levels and the other variables could predict depression, we performed logistic regression analyses with depression/no depression as dependent variable. Patients were allocated to the depression group if they had been given depression as a primary or secondary diagnosis, or the no depression group if they had not been given a depression analysis.

Due to multiple statistical analyses, 0.01 was selected as significance level. In addition, false detection rate adjusted *p*-value (FDR-p) was calculated and applied to all analyses related to the cytokines. IBM Statistical Package for the Social Sciences, Version 23 (SPSS Inc., Chicago, Illinois, USA) software was used in the statistical analysis.

A small group of patients had no or very low levels of certain cytokines (which is a common finding for most cytokines in healthy adults), but the actual value could not be computed by the instrumentation; they are so-called non-detects (NDs). Accordingly, data from the NDs could hold valuable information, and in order to include them in the statistical analyses, we did single imputations, i.e. the NDs were substituted with a random value between zero and the lower detection limit, with a uniform distribution, using the random number generator of SPSS [16].

## Results

### Population characteristics

Population characteristics are presented in Table 1.

**Table 1:** In-patients’ characteristics

Characteristics	
Age, median/SD (years)	76/7.3
80 years and older (%)	39.8
Women (%)	61.2
Men (%)	38.8
Length of stay, median/SD (days)	34/25
Living alone (%)	53
Previous hospitalization (%) ^a^	49
Two or more previous hospitalizations (%) ^a^	38
No known somatic disease (%)	21
Cardiovascular disease (%)	28
Pulmonary disease (%)	10
Thyroid disease (%)	10
Previous stroke (%)	10
Rheumatic disease (%)	3.5
Other somatic diseases (%)	17.5
Potentially anti-inflammatory drug (%) ^b^	55.1
Daily smokers (%)	29.6
BMI, median/SD (kilos)	24/5.3

^a^ Psychiatric hospitalization

^b^ The most common drug in this category is acetylsalicylic acid in low dose as prevention of cardiovascular events (*N* = 30/68.2%)

### Diagnoses

The main diagnostic groups are presented in Table 2. The most common diagnosis was Recurrent depressive disorder (26.5%), the second most common was dementia in Alzheimer’s disease (20.4%). Considering depression as a separate clinical entity, depending on whether the patients had been given depression as a primary or secondary diagnosis or not, the majority of patients could be allotted to the depression group, see Table 2. Selected features of the groups depression and no depression are presented in Table 3.

**Table 2:** Distribution of diagnoses

Diagnoses	ICD-10	%
Organic, including symptomatic, mental disorders	F00–09	37.8
Mental and behavioural disorders due to psychoactive substance abuse	F10–19	1
Schizophrenia, schizotypal and delusional disorders	F20–29	12.2
Affective disorders	F30–39	41.8
Neurotic, stress-related and somatoform disorders	F40–48	7.2
Depression/No depression		60.2/39.8

**Table 3:** Selected features of depressed and non-depressed patients

Comorbidities/features	Depression (%)	No depression (%)
Somatic disease (any)	76.3	82.1
Cardiovascular disease	15.3	46.2
Dementia	22.0	51.3
Antidepressants	74.6	25.6

### Distribution and correlation analysis

The cytokine values of the patients are presented in Table 4. In this group of diagnostically unselected elderly in-patients, no statistical correlation or unequal distribution was found between cytokines and gender, age, BMI, anti-inflammatory drugs, psychotropic drugs, reason for admittance, smoking, vitamin supplements, alcohol consumption, length of stay, somatic disease (present/not-present) or psychiatric diagnoses, dementia included. However, a correlation (FDR-*p* < 0.0044) was found between *no depression* (*N* = 39) and raised levels of several cytokines, see Table 5.

**Table 4:** Serum levels of cytokines (pg/ml) in elderly psychiatric in-patients

Cytokine	Median	SD*	Minimum	Maximum	P 25**	P 75***
IL-1b	3.00	4.65	0.53	38.00	1.58	5.00
IL-1ra	158.00	769.64	31.00	7396.00	84.00	268.75
IL-2	9.00	17.97	0.01	147.00	3.00	16.00
IL-4	3.00	2.55	1.00	13.00	2.00	4.00
IL-5	5.00	5.65	0.73	28.00	3.00	9.00
IL-6	11.00	15.39	3.00	119.00	7.00	18.75
IL-7	21.00	22.83	0.25	104.00	11.00	36.00
IL-8	13.00	9.35	3.00	47.00	8.00	19.00
IL-9	18.00	47.03	3.00	441.00	11.00	28.00
IL-10	8.50	16.92	0.01	102.00	2.00	16.00
IL-12	27.00	51.60	0.05	381.00	11.00	46.75
IL-13	7.00	15.51	0.50	96.00	4.00	14.00
IL-17	42.50	66.57	0.35	371.00	15.25	88.25
Eotaxin	93.00	257.20	28.00	2286.00	65.50	157.75
bFGF	46.00	48.58	1.16	259.00	21.00	79.75
G-CSF	52.00	52.92	1.53	316.00	29.25	82.75
INF-g	184.00	217.26	20.00	1179.00	94.75	280.75
IP-10	1015.50	744.58	216.00	5075.00	762.25	1338.00
MCP-1	19.00	16.04	4.00	128.00	13.25	26.00
MIP-1a	10.00	9.82	2.00	58.00	6.00	15.00
MIP-1b	44.00	24.77	17.00	198.00	34.00	56.00
PDGF-BB	137.50	325.59	1.03	1651.00	34.25	308.00
RANTES	6642.00	12,393.18	532.00	60,319.70	2876.00	16,859.75
TNF-a	92.00	137.85	8.00	1173.00	43.50	127.25
VEGF	21.50	25.94	1.90	130.00	10.00	36.00

* Standard deviation. ** 25-percentile. *** 75-percentile

**Table 5:** Correlation between cytokines and *No depression* (*p*-level > 0.01 excluded)

Cytokine	Correlation coefficient*	p	FDR-p**	Significant***	Eta^2^****
IL-2	0.268	0.008	0.0060	No	0.072
bFGF	0.271	0.008	0.0056	No	0.074
IL-1ra	0.271	0.008	0.0052	No	0.074
IL-1b	0.283	0.005	0.0048	No	0.080
IL-5	0.288	0.004	0.0044	Yes	0.083
IL-12	0.289	0.004	0.0040	Yes	0.083
IL-6	0.290	0.004	0.0036	Yes	0.084
TNF-a	0.295	0.004	0.0028	Yes	0.087
IL-7	0.306	0.002	0.0024	Yes	0.093
IL-10	0.321	0.001	0.0020	Yes	0.103
G-CSF	0.322	0.001	0.0016	Yes	0.104
INF-g	0.334	0.001	0.0012	Yes	0.111
IL-4	0.348	0.001	0.0008	Yes	0.121
IL-8	0.349	0.000	0.0004	Yes	0.122

^*^Spearman Rho; ^**^False detection rate adjusted *p*-value, q-level 0.01, based on *p*-values of all 25 cytokines; ^***^FDR-adjusted p-criterion of 0.0044 ^****^Effect size. Based on Mann-Whitney Z-statistics of all 25 cytokines

While none of the raised cytokine levels predicted depression in a logistic regression model, cardiovascular disease (CVD) and dementia were predictors of patients being categorized as not depressed/no depression (Table 6). However, none of the cytokines came out as predictive in logistic regression models with CVD/no CVD, or dementia/no dementia, as dependent variables.

**Table 6:** Logistic regression model assessing predictors of patients categorized as *no depression*^a^

Predictors of *no depression*	Log odds	SE^b^	Wald Chi^2^	*P*-value	Odds ratio	95% CI^c^ for OR
						Lower - Upper
Dementia (*N* = 20)	1.422	0.484	8.651	0.003	4.147	1.607–10.700
Cardiovascular disease (*N* = 18)	1.664	0.515	10.441	0.001	5.279	1.924–14.479
Constant	−1.409	0.344	16.745	0.000	0.244	

^a^Nagelkerke R Square 0.253, Cox & Snell R Square 0.187

^b^SE: Standard error

^c^CI: Confidence interval

Looking at distributional data, CVD and dementia were more prevalent in the no depression group, compared to the depression group, 46.2% versus 15.3%, and 51.3% versus 22.0%, respectively.

## Discussion

To our knowledge, this is the first study to explore cytokine levels in diagnostically unselected elderly psychiatric in-patients. Using an immunoassay method, we analysed 27 plasma cytokines in 98 patients, 60 years and older, admitted to a gerontopsychiatric unit.

The results demonstrated that cytokine levels did not correlate with variables such as age, gender, psychiatric diagnoses, somatic disease (present/not present), and the use of anti-inflammatory and psychotropic drugs. Considering each of these factors separately, this does not seem to match previous findings, as prior studies have shown positive associations between for instance Alzheimer’s disease and increased levels of several cytokines [17], and between aging and increased levels of IL-6 and TNF-α [18]. Then again, our heterogeneous study population differs substantially from most of the diagnostically uniform populations previously studied.

The high frequency of somatic and psychiatric comorbidity in the study population may have contributed to the increased levels of cytokines in general, masking possible correlations between any single factor and changes in levels of cytokines. On the other hand, there is a possibility that altered immune activity in psychiatric patients is a general phenomenon, not restricted to specific diagnoses. Such a hypothesis can be bolstered by the fact that research has shown raised levels of inflammatory markers in several psychiatric disorders, ranging from schizophrenia to anxiety disorders [19, 20].

Contrary to some prior studies, we did not find any correlation between cytokines and depression. Given the fact the majority of our depressed patients were diagnosed with recurrent depressive disorder, it could be hypothesized that relapsing depression in the elderly represents a somewhat different immunological process compared to depression in younger patients, on whom most studies have been conducted. On the other hand, our intra-group comparison could be the main explanation why the depressed patients did not have comparably higher levels of cytokines, considering that the overall level of cytokines was high regardless of diagnosis.

One relevant point to consider is that anti-depressants have exhibited anti-inflammatory properties in both clinical and experimental studies [21, 22]. Accordingly, the high percentage of antidepressant use in the depression group (74.6% versus 25.6% in the no depression group) might have been a factor in terms of lowering the cytokine levels amongst the depressed patients, cancelling out the association between depression and cytokine levels.

We did observe a correlation between *no depression*, a term denoting patients without significant depression irrespective of other clinical features, and several cytokines (FDR-*p* < 0.0044). Presumably, this correlation is not explained by the lack of depression per se. Rather, it is more likely that other factors contribute to increased cytokines in the no depression group. CVD and dementia are two possible contributing factors, as they are more prevalent in the no depression group, hence becoming predictors in a binary logistic model. Both CVD and dementia have been linked to increased levels of cytokines [23–26]. Yet, the fact that none of the cytokines correlated with dementia or CVD, in addition to the absence of predictive power of any of the cytokines for both dementia and CVD in binary logistic regression, indicate that other, unrecognized factors also contribute to the association between no depression and elevated cytokines.

Finally, it should be taken into account that the total level of psychiatric morbidity in the no depression group (e.g. organic mental disorders) was probably just as high, perhaps higher, than in the depression group, hence diluting the potential effect of depression on the cytokine levels. There might also be a proportion of the no depression patients with dementia that actually was depressed, as it cannot be ruled out that patients with dementia express depressive symptoms in a way that is less likely to be recognized by the clinician. Then again, altered cytokine levels in psychiatric patients may be a general phenomenon, possibly being primarily dependent on the severity of the disorder, and not the diagnosis.

When interpreting the result of our study, we need to be cautious, given the size of the study, and particularly the size of the subgroups. It should also be noted that though we chose 0.01 as significance levels due to multiple comparisons, and calculated FDR-p for the cytokine statistics, the risk of spurious correlations is still present. On this point, it worth mentioning that a Bonferroni correction would have required a significance threshold of 0.002. Moreover, applying few exclusion criteria may have provided a study population that resembled real-life gerontopsychiatric in-patients, but heightened the risk of confounders due to the general heterogeneity of the group, i.e. differences in age, socioeconomic background, lifestyle factors etc. Adding to this risk was the possibility of greater variability in health status, including immunological functioning, in the elderly compared to younger adults. Another possible source of cytokine variability could be that not all blood samples were fasting (35.7% non-fasting). Though the studies are somewhat conflicting, most seem to indicate that fasting has a certain anti-inflammatory effect [27–29]. A correlational analysis between fasting and cytokine levels in our population demonstrated nevertheless no correlation. Furthermore, a control group of healthy elderly would have made a statistical comparison possible, but at the time, we did not have such data available. Finally, it should be mentioned that single imputation of data to remedy NDs (see section Statistical analysis) may confer a risk of distorting the statistics, in particular when the number of NDs are high. In our study, the number of NDs are small (four at the most) and running the statistics without the NDs did not produce any significant change.

The plasma levels of cytokines observed in this group of elderly patients were the results of complex immunological processes, where different cytokines might have played different roles at different stages. Several interplaying factors, such as age, life style factors, somatic health, genetics, drugs and the psychiatric disorders per se, may have contributed in these processes. Besides, it is still unclear how an increased level of cytokines in systemic circulation relates to neuroimmunological processes in psychiatric disorders. Adding to the complexity is the application of various methods of cytokine analysis and methodological issues [30, 31]. Hence, caution should be exercised when interpreting data and making inferences about cytokine profiles and biomarkers in psychiatry, and perhaps particularly in the elderly population. This also begs the question as to what extent findings in younger adults, with uniform diagnostic profiles and no comorbidity, have bearings on real-life elderly patients.

Though the field of old age neuroimmunology has made great advances in the last decade, there is still a lot to be learned about the immunology of gerontopsychiatry. Further studies are needed on gerontopsychiatric patients in general and gerontopsychiatric patients with specific disorders, preferably with patients that are physically healthy. Clinical studies are necessary to gauge the immunological effects, both cellular and humoral, of various forms of treatment. As for our patients, the question is if psychiatric treatment can impact the high, probably multi-etiological, levels of cytokines. Finally, genomic and proteomic studies are required to uncover the immunological underpinnings of psychiatric disorders affecting the elderly, including longitudinal studies of healthy populations at risk.

## Conclusions

There was no significant difference in cytokine levels between various psychiatric diagnoses in elderly psychiatric in-patients. However, when patients were allocated to two groups, *depression* and *no depression*, irrespective of diagnoses and other clinical features, we found higher levels of certain cytokines in the *no depression* group compared to the *depression* group. This might be due to a higher frequency of CVD and dementia in the *no depression* group, as well as other unknown factors.

## Abbreviations

BMI: Body mass index
CVD: Cardiovascular disease
EDTA: Ethylenediaminetetraacetic acid
FDR: False detection rate
ICD-10: International classification of diseases version 10
NDs: Non-detects
SPSS: Statistical package for the Social Sciences
